# The magnitude and cross reactivity of SARS-CoV-2 specific antibody responses in Sri Lankan children and association with the nutritional status

**DOI:** 10.1186/s12879-025-11967-3

**Published:** 2025-11-04

**Authors:** Chandima Jeewandara, Maneshka Vindesh Karunananda, Suranga Fernando, Saubhagya Danasekara, Gamini Jayakody, S. Arulkumaran, N. Y. Samaraweera, Sarathchandra Kumarawansha, Subramaniyam Sivaganesh, P. Geethika Amarasinghe, Chintha Jayasinghe, Dilini Wijesekara, Manonath Bandara Marasinghe, Udari Mambulage, Helanka Wijayatilake, Kasun Senevirathne, A. D. P. Bandara, C. P. Gallage, N. R. Colambage, A. A. Thilak Udayasiri, Tharaka Lokumarambage, Y. Upasena, W. P. K. P. Weerasooriya, Tiong Kit Tan, Alain Townsend, Graham S. Ogg, Gathsaurie Neelika Malavige, the seroprevalence study group

**Affiliations:** 1 Allergy Immunology and Cell Biology Unit, Department of Immunology and Molecular Medicine, Faculty of Medical Sciences, University of Sri Jayewardenepura, Nugegoda, Sri Lanka; 2 Ministry of Health, Colombo, Sri Lanka; 3 MRC Translational Immune Discovery Unit, MRC Weatherall Institute of Molecular Medicine, University of Oxford, Oxford, UK; 4 Chinese Academy of Medical Sciences Oxford Institute, University of Oxford, Oxford, UK

**Keywords:** SARS-CoV-2, Antibodies, Seroprevalence, Variants, Body mass index, ACE2 blocking antibodies

## Abstract

**Background:**

In order to determine if undernutrition affects the presence, breadth and magnitude of antibodies to SARS-CoV-2 and variants, we studied SARS-CoV-2 specific antibody responses in a large island wide serosurvey in children in Sri Lanka.

**Methods:**

Using the WHO UNITY protocol, we recruited 5207 children, aged 10 to 20 years, and assessed anthropometric measures, seropositive rates, ACE2 blocking antibodies and antibodies to omicron variants, in vaccinated and unvaccinated children.

**Results:**

3111/3119 (99.7%) vaccinated and 2008/2088 (96.2%) of unvaccinated children were seropositive for SARS-CoV-2, although the detection of ACE2 blocking antibodies were significantly higher in vaccinated children (2984/3111, 95.9%) compared to unvaccinated (1346/2008, 67.0%). 1057 (22.1%) had a BMI < 3rd centile for age, and therefore were classified as underweight. Unvaccinated children, with < 3rd BMI centile had significantly lower ACE2 blocking antibodies than other groups. There were no differences in the antibody titres to XBB.1.5 or BA.2.75 based on the BMI category.

**Conclusions:**

The high seropositivity rates, with high antibody titres to SARS-CoV-2 variants in unvaccinated children indicates possible multiple infections with SARS-CoV-2. The implications of lower antibody levels in underweight children should be further investigated.

**Supplementary Information:**

The online version contains supplementary material available at 10.1186/s12879-025-11967-3.

## Background

Despite high vaccination rates and high seroprevalence rates in many countries, outbreaks of COVID-19 still occur in many regions, with the JN.1, BA.2, BA.2.86 and their sub-lineages dominating [1, 2]. Although the number of hospital admissions and case fatality rates are low, COVID-19 still causes a significant impact on health care systems in some countries [1]. Many high-income countries and some lower-middle income countries are administering booster doses to their populations with updated versions of the COVID-19 vaccines, incorporating the XBB.1.5 variant in 2023 [3]. While many countries are making these updated COVID-19 booster doses only available to vulnerable individuals, the CDC in USA has recommended these vaccines to all individuals above the age of six months [3].

Sri Lanka experienced many COVID-19 outbreaks in the past, with high mortality rates, especially during the outbreak due to the delta variant [4]. However, deaths have been predominantly among the adults, with children rarely developing severe disease, as seen in all other countries [5]. Although 28.5% of the population in Sri Lanka are children (< 18 years of age), they accounted for < 18% of reported cases of COVID-19, possibly due to the asymptomatic nature of infection among children [6]. Furthermore, unlike many other countries globally and in the region, Sri Lanka did not offer any bivalent booster doses for individuals in Sri Lanka and children >12 years of age were offered only two doses of the Pfizer BioNTech (BNT162b2) vaccine.

The nutritional status affects immunity to many viral infections, and the intake of micro and macronutrients has shown to affect susceptibility to SARS-CoV-2 infection [7]. Obesity has shown to be associated with a significantly lower antibody responses to COVID-19 vaccines [8, 9]. However, there are limited data on antibody responses to SARS-CoV-2 following natural infection or vaccination in those who are underweight or malnourished. As Sri Lanka is going through an economic crisis, it has been estimated that 28.2% of the population are below the poverty line (< US$ 3.65 per day) in 2023 with 2.9 million children in urgent need of humanitarian assistance [10]. Therefore, it would be important to determine if undernutrition affects the antibody levels to SARS-CoV-2 and the breadth of responses. This will enable us to understand the extent of population immunity that will affect transmission dynamics when novel variants are introduced into the population.

In this study, to understand the immunity to SARS-CoV-2 in the population and to investigate the association of antibody responses in underweight children in those with normal nutrition status, we measured the presence, breadth and the magnitude of antibodies to SARS-CoV-2 during an island wide serosurvey using the WHO UNITY protocol.

## Materials and methods

### Study participants and sampling technique

**Fig. 1:**
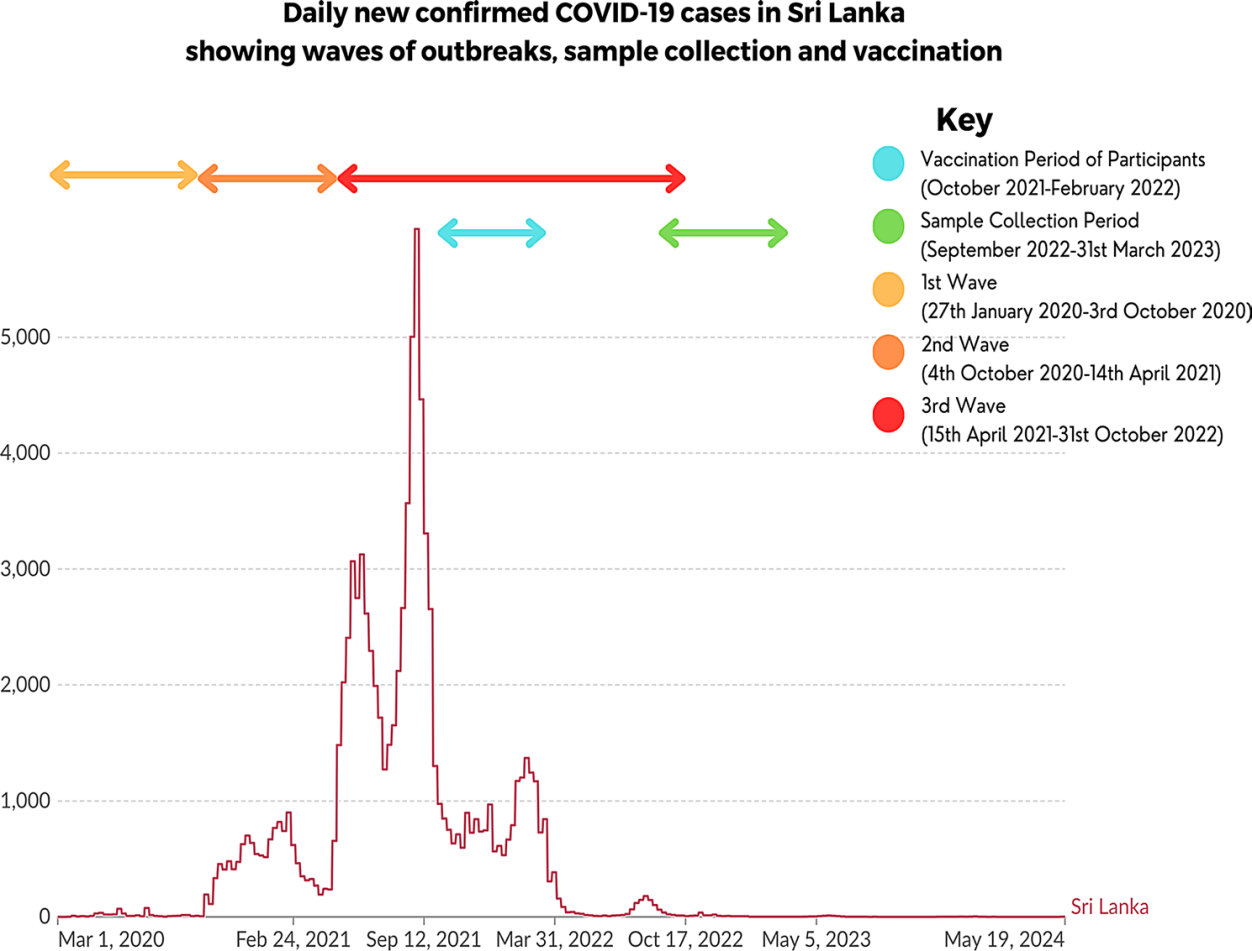
The timing of vaccination campaigns and waves of variants in Sri Lanka is shown in relation to recruitment of children into the period of study from September 2022 to March 2023. The first wave in Sri Lanka was seen due to the B.1.411 until the end of March 2021, when the alpha variant (B.1.1.7) emerged and became the dominant lineage (second wave), immediately followed by the delta variant [29], which was the third wave. The figure was adapted by data presented in our world in data [1]

We recruited 5207 school children between the age of 10 to 20 years, who were attending public or private schools in Sri Lanka, during September 2022 to 31st March 2023 as previously described according to the WHO UNITY protocol [11]. The timing of recruitment of children in relation to different waves in Sri Lanka and administration of vaccines is shown in Fig. 1. Briefly, children were recruited following informed written consent from the parents/guardians and assent was taken from children. The study was carried out in nine districts in Sri Lanka, representative of each of the nine provinces. A stratified multi-stage cluster sampling method was used to select the schools in each district, with a cluster size of 40 students from each cluster. A probability proportionate to the size (PPS) sampling technique was used to select the sample size from each district, as the population size and urbanicity grade varied in different districts. A pre-tested and structured interviewer-administered questionnaire was used to record basic demographic details and details of the vaccination history (supplementary questionnaire).

### Ethics statement

The study was approved by the Ethics Review Committee of the University of Sri Jayewardenepura, Sri Lanka (Ethics approval number: COVID 13/21) and also received administrative clearance of the Ministry of Health, Sri Lanka. All subjects and their parents/guardians gave informed written consent.

### Assays for SARS-CoV-2 specific total antibodies and ACE2 blocking antibodies

SARS-COV-2 specific total antibody (IgM, IgG and IgA) responses against the receptor binding domain of the spike protein were evaluated using the Wantai SARS-CoV-2 Ab ELISA (Beijing Wantai Biological Pharmacy Enterprise, China) as previously described. This assay has been used in carrying out serosurveys for SARS-CoV-2 previously in Sri Lanka and was shown to have a sensitivity of 98% and a specificity of 100% using pre-COVID-19 era samples [12]. The antibody index was calculated by dividing the absorbance of each sample by the cut-off value, according to the manufacturer’s instructions.

The ACE2 blocking antibodies were measured in all samples that tested positive by the SARS-COV-2 specific total antibody (IgM, IgG and IgA), using the surrogate Nab test (sVNT, Genscript Biotech, USA) that been widely used as a surrogate measure for the presence of neutralizing antibodies (Nabs) including previous studies in Sri Lanka [13, 14]. An inhibition percentage ≥ 25% was considered as positive for ACE2 blocking antibodies [14]. The sVNT assay was only conducted for samples that gave a positive result with the Wantai SARS-CoV-2 Ab ELISA.

### Haemagglutination test (HAT) to detect antibodies to the receptor binding domain (RBD) of omicron variants

The HAT was carried out as previously described using the BA.2.75 and XBB.1.5 versions of the IH4-RBD reagents with additional mutations in the RBD (Y365F, T392W and V395I) [15, 16], as these were the predominantly circulating SARS-CoV-2 variants in 2023. The assays were carried out and interpreted as previously described by us at serum dilutions of 1:40 and 1:80 in serosurveys carried out in the Colombo district in Sri Lanka [17]. A titre of 1:40 was considered as a positive response, as previously described [18]. A HAT titre of 1: 40 was shown to detect 99% of samples which had neutralizing antibody titres of ≥ 20 (50% inhibitory concentrations, IC_50_) assessed with the microneutralization assay [18].

### Assessment of the body mass index

The height was measured by a stadiometer to within 0.5 cm and weight was measured using a digital scale, which was calibrated regularly throughout the study. The BMI centile was derived by plotting the values on the WHO BMI for age growth charts for boys or girls to acquire the percentile ranking, as this was shown to be the most suitable indicator for growth patterns in children [19]. The BMI centile for age was used instead of Z-score for BMI for age, as it was shown to overestimate the proportion of children with malnutrition in some populations [20].

### Statistical analysis

GraphPad Prism version 10.1 was used for statistical analysis. As the data were not normally distributed, differences in means were compared using the Mann-Whitney U test (two tailed), and the Kruskal-Wallis test was used to compare the differences of the antibody levels between vaccinated and unvaccinated children in the different districts. A post hoc analysis for the relationship between BMI centiles, in different districts and urbanicity was carried out using the chi square test setting the standardized residual value at 0.05 significance level, with the alpha level adjusted by using Bonferroni correction.

## Results

### Vaccination uptake rates in children in different districts in Sri Lanka

3119/5207 (59.90%) of children had received at least one dose of the COVID-19, Pfizer BioNTech (BNT162b2) vaccine and 1967/5207 (37.78%) had received two doses. Of those who were eligible to take the vaccine (children ≥ 12 years of age), the overall vaccination rates were 3086/4155 (74.27%). None of the children had received any booster doses, as these were not made available to children under the age of 19 years. The number of children in each age group in each district, who were vaccinated and unvaccinated along with seropositivity rates are shown in supplementary Table 1. The positivity rates for ACE2 blocking antibodies of vaccinated children and unvaccinated children are shown in supplementary Table 2. The seropositivity rates in vaccinated and unvaccinated children in each age group in each district is shown in supplementary Tables 3 to 11 and the ACE2 blocking antibody positivity in vaccinated and unvaccinated children in each age group in different districts is shown in supplementary Tables 2 to 20.

3111/3119 (99.7%) children who had received at least one dose of the vaccine were seropositive for SARS-CoV-2 and 2008/2088 (96.2%) of unvaccinated children (Supplementary Table 1). There was no difference in the seropositivity rates in unvaccinated children in urban (97.5%), rural (95.8%) and estate (96.2%), showing that children in all areas in Sri Lanka were equally infected with the SARS-CoV-2 virus. 2984/3111 (95.9%) children who had received at least one dose of the vaccine had ACE2 blocking antibodies above the cut-off threshold of a positive response compared to 1346/2008 (67.0%) of unvaccinated children. Unvaccinated children had significantly lower (*p* < 0.0001) titres than vaccinated children. The positivity rates for ACE2 blocking antibodies were significantly higher in unvaccinated children in urban (291/391, 74.4%, *p* = 0.0016) and estate areas (37/48, 77.1%, *p* = 0.004), compared to children living in rural areas (1018/1569, 64.9%).

We carried out HAT assays to assess antibody responses to BA.2.75 and XBB.1.5 in samples of 10% of the unvaccinated and vaccinated seropositive children, which were randomly selected representative of all the nine districts (*n* = 202). 130/202 (64.3%) vaccinated and 55 (27.2%) unvaccinated children had an antibody titre of ≥ 1:40 to BA.2.75. For XBB.1.5, 87 (43.1%) vaccinated and 62 (30.7%) unvaccinated children had an antibody titre of ≥ 1:40.

### The magnitude of antibody responses to SARS-CoV-2 based on the body mass index

As previously described by us [21], in this island wide large cohort of children, 4782/5207, were children between the ages of 10 to 18, and the BMI centile was used as an surrogate indicator of their nutritional status in this age group. In this cohort of children aged 10–18 (*n* = 4782), 1057 (22.1%) had a BMI < 3rd centile for age, and therefore were classified as underweight. 215 (4.5%) children had a BMI of >97th centile for age, and were considered severely overweight. The BMI centiles significantly varied among the different districts (chi square 159.4, *p* < 0.001) with Gampaha district with predominantly urban areas having a higher proportion of children with BMI centiles >97th, while Matara district with many rural areas had a higher proportion of children with a BMI centile < 3rd.

**Fig. 2:**
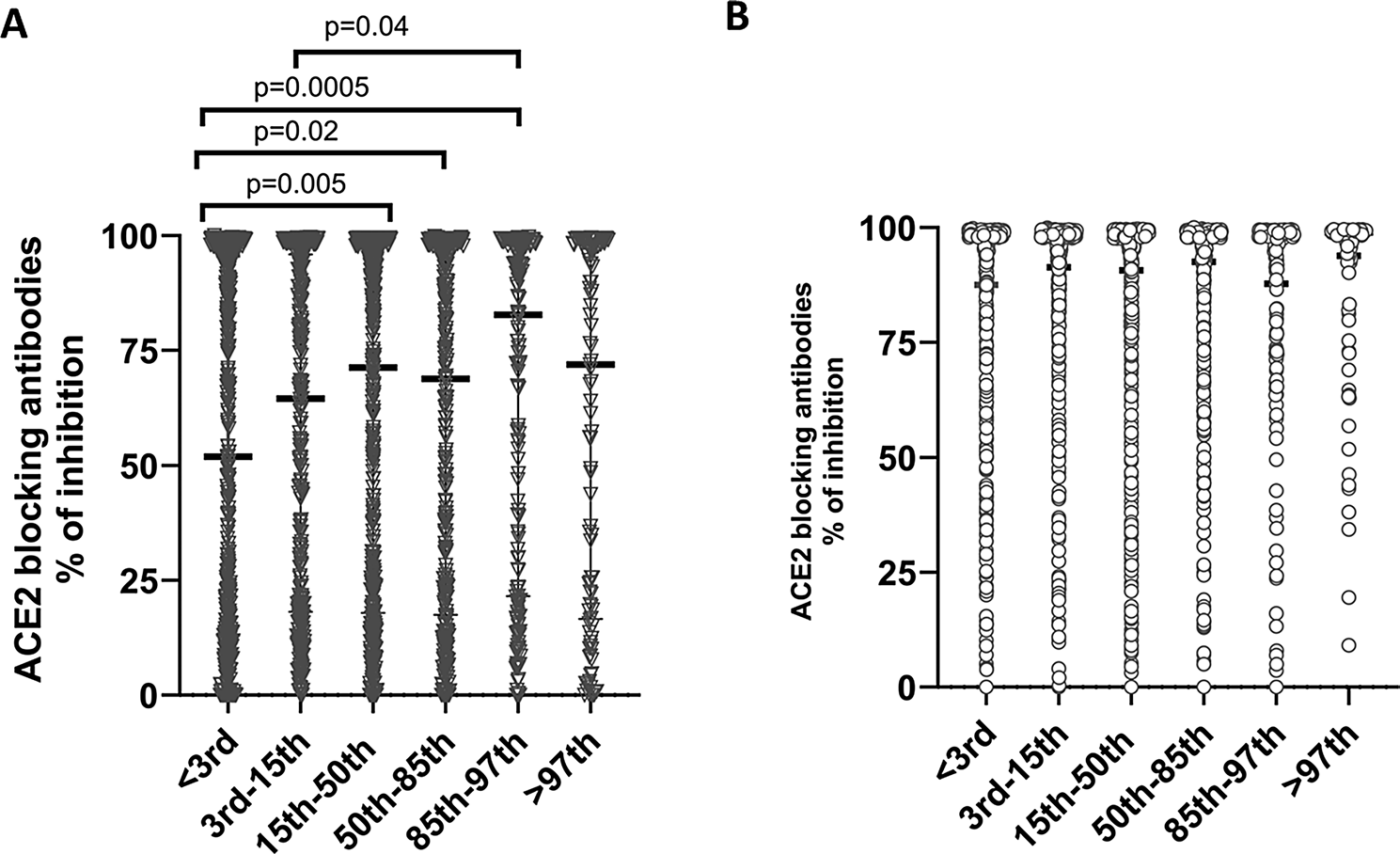
ACE2 blocking antibody levels determined by the surrogate SARS-CoV-2 neutralising antibody assay (sVNT) in vaccinated and unvaccinated children of different BMI centile categories. The ACE2 blocking antibody levels (% of inhibition) were measured in unvaccinated (A, *n* = 1973) and vaccinated (B, *n* = 2723) children aged 10–18 years of age, who tested seropositive by the SARS-CoV-2 total antibody assay. The ACE2 blocking antibody titres were compared between children of different BMI centiles. The Mann-Whitney U test (two tailed) was used to calculate the differences in means of ACE2 antibody titres in different BMI groups in the vaccinated and unvaccinated groups. All tests were two sided. Data are presented as median values +/- interquartile ranges as appropriate

Among the unvaccinated children, those who had a BMI of < 3rd centile had significantly lower ACE blocking antibodies (median 51.9, IQR 13.1 to 95.5% of inhibition), which is a surrogate marker for the presence of Nabs compared to children of other categories (Fig. 2A). Children with a BMI centile between 85th to 97th had the higher titres of ACE2 blocking antibodies (median 82.7, IQR 21.6 to 99.7, % of inhibition). Among vaccinated children the ACE2 blocking antibody titres were similar in children of different BMIs (Fig. 2B). There were no differences in the antibody titres to XBB.1.5 or BA.2.75 based on the BMI category.

## Discussion

In this study we assess the relationship between the BMI and the presence, breadth and the magnitude of antibodies to SARS-CoV-2 during an island wide serosurvey among vaccinated and unvaccinated children, representing all the nine provinces in Sri Lanka. We found that the overall seropositivity rates of the unvaccinated children (96.2%) and vaccinated children (99.7%) were similar, indicating a high infection rate in all areas in Sri Lanka by March 2023. Overall, 67.0% of unvaccinated children had ACE2 blocking antibody titres above the cut-off threshold and the median values were 67.6%, which were several folds higher than what we found in individuals who had one natural infection with SARS-CoV-2 in 2020 [14]. Therefore, given the high positivity rates for ACE2 blocking antibodies and high titres seen in unvaccinated children, it is likely that children (vaccinated and unvaccinated) are likely to have been infected more than once with SARS-CoV-2.

We assessed antibody responses to omicron sub-lineages BA.2.75 and XBB.1.5 in the sub cohort of vaccinated and unvaccinated children. 64.3% of vaccinated and 27.2% unvaccinated children had antibody titres above the positive cut off threshold to BA.2.75, and 43.1% of vaccinated and 30.7% unvaccinated children to XBB.1.5. Sri Lanka reported circulation of BA.2.75 variants during the latter part of 2022 [2], while the XBB variants were only found after the study recruitment had finished. Therefore, although the children could have experienced infection with BA.2.75, they are less likely to have been exposed to the variants of the XBB lineage. Interestingly, the vaccinated children had significantly higher antibody responses to BA.2.75 than to XBB.1.5. As XBB.1.5 has many more mutations within the RBD than BA.2.75 [22], it is likely that it escapes vaccine induced immunity to a far greater extent than BA.2.75. Furthermore, many children received their vaccines during the BA.2 wave in Sri Lanka [2], it is likely they would have been exposed to the vaccine virus and BA.2 during a very short period, thereby inducing robust immune responses to BA.2 sub-lineages.

Sri Lanka has been going through an economic crisis, with many children not having access to sufficient nutritious food for the past 2 or 3 years due to high inflation rates resulting in food prices soaring [10]. Indeed, we found that 22.1% of children were < 3rd BMI centile for age, indicating under nutrition. There were significant differences in ACE2 blocking antibody levels among unvaccinated children in different BMI groups, which are surrogate markers of Nabs [14, 23]. Nabs antibodies prevent binding to the ACE2 receptor and have shown to associate with protection [24]. Children with undernutrition (< 3rd BMI centile for age), had significantly lower ACE2 blocking antibody titres compared to children of healthy weight. However, in this study we only investigated antibody responses to the spike protein using different types of assays and it would be important to understand the antibody responses to other proteins such as the N protein, which has also shown to associate with protection. Furthermore, although more recent SARS-CoV-2 omicron variants almost completely evade neutralization with antibodies specific to the earlier SARS-CoV-2 variants (Wuhan-Hu-1) [22], they do not completely evade T cell responses [25, 26]. Previous studies in South Africa have shown that children who were malnourished or had some indicator of diminished growth had lower antibody titres to measles and tetanus [27]. Furthermore, another study in Ecuador showed that underweight children had significantly lower antibody titres to the tetanus toxoid, rotavirus and respiratory syncytial virus, while anaemic children had antibody titres below the protective threshold for diphtheria [28]. Therefore, to fully understand the population immunity to SARS-CoV-2 variants, also in the context of nutrition status, it would be important to assess the functionality, magnitude and breadth of T and B cell responses to the virus.

## Conclusions

Underweight unvaccinated children were more likely to have lower SARS-CoV-2 antibody responses, compared to children with a normal weight. The implications in regard to protection from SARS-CoV-2 should be further investigated. In addition, in the context of the nutrition status, it would be important to assess the functionality, magnitude and breadth of T and B cell responses to the virus.

## Supplementary Information

Below is the link to the electronic supplementary material.

Supplementary Material 1

Supplementary Material 2

## Data Availability

Data is provided within the manuscript or supplementary information files.

## Abbreviations

ACE2: Angiotensin-converting enzyme 2
BMI: Body mass index
HAT: Haemagglutination test
RBD: Receptor binding domain
sVNT: Surrogate virus neutralization test
WHO: World Health Organization
