# Supplementary material for: The magnitude and cross reactivity of SARS-CoV-2 specific antibody responses in Sri Lankan children and association with the nutritional status
Source: BMC Infect Dis. 2025 Nov 4;25:1497. doi: 10.1186/s12879-025-11967-3 (PMC12584496; doi:10.1186/s12879-025-11967-3)
Supplement: Supplementary file 2 — Supplementary Material 2 [file 12879_2025_11967_MOESM2_ESM.pdf]

Annex I

Data Collection Form

Investigating the Seroprevalence of SARS-CoV-2 Infections and Dengue Infections in Sri Lankan Children

Name:

DOB:

Address:

Name Code:

Telephone:

Date:

School:

School Code:

1. **Sex:**

Male		Female	

2. **Height:** cm
3. **Weight:** kg
4. **Waist Circumference** cm

Questions to be asked from the parent/guardian:

5. Has your child been vaccinated against COVID-19?

	Yes		No

6. If yes, please provide the following details:

	First Dose				Second Dose			
Type of Vaccine	Yes	No	Don't Know	Date of Vaccination	Yes	No	Don't Know	Date of Vaccination
Pfizer								
Sinopharm								
AstraZeneca								
Moderna								
Sputnik V								

Third Dose: Given/Not Given

Type of Vaccine:…………………………………………………………………………

7. Has your child had COVID-19 infection during the last two years?

	Yes		No		Don’t Know

If the answer is “No”, please go to question number **13**

8. If yes, how was it confirmed?

**Test**	**Yes**	**No**	**Don’t Know**	**Date of Test**
PCR				
RAT				

9. Did the child have any symptom of COVID-19?

	**Yes**	**No**	**Don’t Know**
a. A few days before the diagnosis			
b. Until 14 days after the diagnosis			

10. What was the level of care offered for the child?

	**Yes**	**No**	**Don’t Know**
a. Home-based care			
b. Intermediate Care Centre			
c. COVID-19 Treatment Hospital			
d. HDU or ICU Care			

11. If institutionalized, what was the reason for institutionalization?

	**Yes**	**No**	**Don’t Know**
a. It was the policy of the government to hospitalize everyone			
b. The child was hospitalized as parents/care givers were worried about COVID-19			
c. The child required hospitalization as the child was ill			

12. Details of hospital stay **[To be filled with data extracted from the diagnosis card]**

a. What is the diagnosis written in the card?	
b. What was the recorded lowest oxygen saturation level?	
c. Was the child given oxygen at any time point?	
d. Did the child need ICU admission?	

13. Immunization history of the child

Vaccine	Yes	No	AEFI (if observed)
BCG			
OPV 1			
DPT 1			
Hep B 1			
Hib 1			
fIVP 1			
OPV 2			
DPT 2			
Hep B 2			
Hib 2			
fIVP 2			
OPV 3			
DPT 3			
Hep B 3			
Hib 3			
MMR 1			
JE			
OPV 4			
DPT 4			
MMR 2			
OPV 5			

DT			
HPV 1			
HPV 2			
aTd			

Past history of dengue infection (to be retrieved from medical records)

1. Has your child ever had confirmed dengue infection?

	Yes		No		Don't Know

If the answer is "No", please go to the end of the questionnaire

2. If the answer is yes, when was it?

3. What was the level of care offered for the child?

	**Yes**	**No**	**Don't Know**
a. Home-based care			
b. Hospital general medical/pediatric ward care			
c. Hospital high dependency (HDU) care			
d. Hospital intensive (ICU) care			

4. Data on investigations and clinical outcome

	**Yes**	**No**	**Don't Know**
a. Thrombocytopenia			
If yes, what was the lowest platelet count			
b. Was an Ultrasound Scan of the abdomen done?			
c. Did the child have pleural effusion/ascites (Confirmed by an Ultrasound Scan)			
d. Did the child have any bleeding manifestations?			
If yes, describe			

e. Did the child have Dengue Haemorrhagic Fever/Dengue Shock Syndrome (based on the diagnosis entered on the diagnosis card)?			
